# The impact of preoperative left atrial appendage thrombus on surgical outcomes in atrial fibrillation patients undergoing epicardial left atrial appendage closure

**DOI:** 10.3389/fcvm.2026.1779256

**Published:** 2026-05-14

**Authors:** Qing Yao, Fei Liu, Yu Yang, Dong Xu

**Affiliations:** Department of Cardiac and Vascular Surgery, Beijing Tiantan Hospital, Capital Medical University, Beijing, China

**Keywords:** atrial fibrillation, classification, left atrial appendage closure, left atrial appendage thrombus, surgery

## Abstract

**Background:**

The presence of a left atrial appendage (LAA) thrombus is traditionally considered a contraindication to epicardial left atrial appendage closure (LAAC) because of the risk of periprocedural embolization. This presents a significant challenge for atrial fibrillation (AF) patients with a high bleeding risk who are intolerant to long-term anticoagulant treatment. The safety and outcomes of epicardial LAAC in patients with a preexisting LAA thrombus remain poorly defined.

**Methods:**

This retrospective cohort study included 108 AF patients who underwent epicardial LAAC at a single center. Patients were stratified into two groups on the basis of preoperative imaging: the Thrombus Group (*n* = 11) and the No-Thrombus Group (*n* = 97). Intraoperative management, perioperative complications, and long-term clinical outcomes (cerebrovascular events and all-cause mortality) were compared between the groups. An imaging-based classification of thrombus morphology (Types I-III) was proposed for risk stratification.

**Results:**

No intraoperative strokes or deaths occurred in either group. Early postoperative complication rates were low and not significantly different between groups. During follow-up, the rates of thromboembolic events (1.0 vs. 2.3 per 100 patient-years) and all-cause mortality (0.3 vs. 0.0 per 100 patient-years) were comparable between the No-Thrombus and Thrombus groups, with no statistically significant differences. Thrombi confined to the LAA tip (Type I) were associated with favorable outcomes.

**Conclusion:**

Epicardial LAAC appears to be a feasible and safe alternative for stroke prevention in some AF patients with LAA thrombi who are unsuitable for anticoagulation therapy, as it does not increase perioperative risk or compromise long-term treatment efficacy.

## 1 Introduction

Atrial fibrillation (AF) is the most common sustained cardiac arrhythmia worldwide and constitutes a major global health burden because of its strong association with an increased risk of thromboembolic stroke (1). It is estimated that AF is responsible for 15%–20% of all ischemic strokes (2). These strokes are often more severe, lead to greater disability, and have higher mortality rates than strokes from other causes do (3).

The vast majority of thrombi in nonvalvular AF originate from the left atrial appendage (LAA) (4). The LAA is a small, blind-ended, and trabeculated pouch derived from the embryonic left atrium and is a primary site for blood stasis and thrombus formation in the context of disordered mechanical activity during AF (5, 6). Therefore, the LAA is a key therapeutic target for stroke prevention (7).

Percutaneous and epicardial left atrial appendage closure (LAAC) have emerged as effective strategies for stroke prevention in patients with AF who are unsuitable for long-term oral anticoagulant treatment (8, 9). By physically excluding the LAA from the systemic circulation, these procedures aim to eliminate the primary source of cardioembolic strokes (10). The safety and efficacy of LAAC are well established, leading to its incorporation into major international guidelines (11).

The presence of a LAA thrombus, typically identified by preprocedural imaging such as transesophageal echocardiography (TEE), transthoracic echocardiography (TTE), or cardiac computed tomography angiography (CTA), has traditionally been considered a contraindication to percutaneous or epicardial LAAC (12). This is because device manipulation may dislodge the thrombus, potentially causing a catastrophic periprocedural embolic event. Consequently, current guidelines mandate a period of anticoagulant treatment to resolve the thrombus before proceeding with the surgery (4, 13). However, this approach presents a significant challenge for patients with a high bleeding risk who are intolerant to anticoagulant treatment, such as those with a history of intracranial hemorrhage, gastrointestinal ulcer bleeding, or urinary tract bleeding, as long-term anticoagulant treatment may itself lead to life-threatening hemorrhagic events (14). Therefore, for anticoagulant treatment-intolerant AF patients undergoing epicardial LAAC as a standalone procedure, the optimal management strategy and the associated risks in the context of an incidentally identified LAA thrombus remain poorly defined (15).

There is a notable paucity of data directly comparing the perioperative and postoperative outcomes of patients with and without a preexisting LAA thrombus who undergo epicardial LAAC. Key questions regarding the impact of a preexisting LAA thrombus on surgical complexity, perioperative complication rates (particularly stroke), and short-term recovery remain largely unanswered.

Therefore, this study aims to elucidate this unresolved issue through a retrospective comparative analysis, specifically evaluating the impact of a preoperatively identified LAA thrombus on intraoperative management, postoperative complications, and clinical outcomes in AF patients who underwent epicardial LAAC.

## 2 Materials and methods

### 2.1 Study design and patient population

This retrospective cohort study was conducted at Beijing Tiantan Hospital and included consecutive patients who underwent epicardial LAAC between October 2019 and November 2025. The study protocol was reviewed and approved by the Institutional Review Board of Beijing Tiantan Hospital (Approval No.: KY2022-013-02), and written informed consent was obtained from all the participating patients.

Eligible patients were adults (age >18 years) with non-valvular AF who underwent epicardial LAAC, either as a standalone totally thoracoscopic procedure or concomitantly with other cardiac surgeries (e.g., off-pump coronary artery bypass). The LAA thrombi were classified into three types according to their morphology and location: Type I—thrombus confined to the tip of the LAA, occupying less than half of the LAA volume; Type II—thrombus extending beyond half of the LAA volume but not reaching the LAA orifice; Type III—thrombus filling the entire LAA and extending beyond the orifice into the left atrial cavity. Patients with Type I LAA thrombus and those without thrombus were included. The exclusion criteria were as follows: (1) incomplete preoperative imaging data; (2) a history of left thoracotomy; (3) valvular AF; and (4) prior cardiac surgery.

### 2.2 Patient stratification and imaging assessment

**Figure 1:**
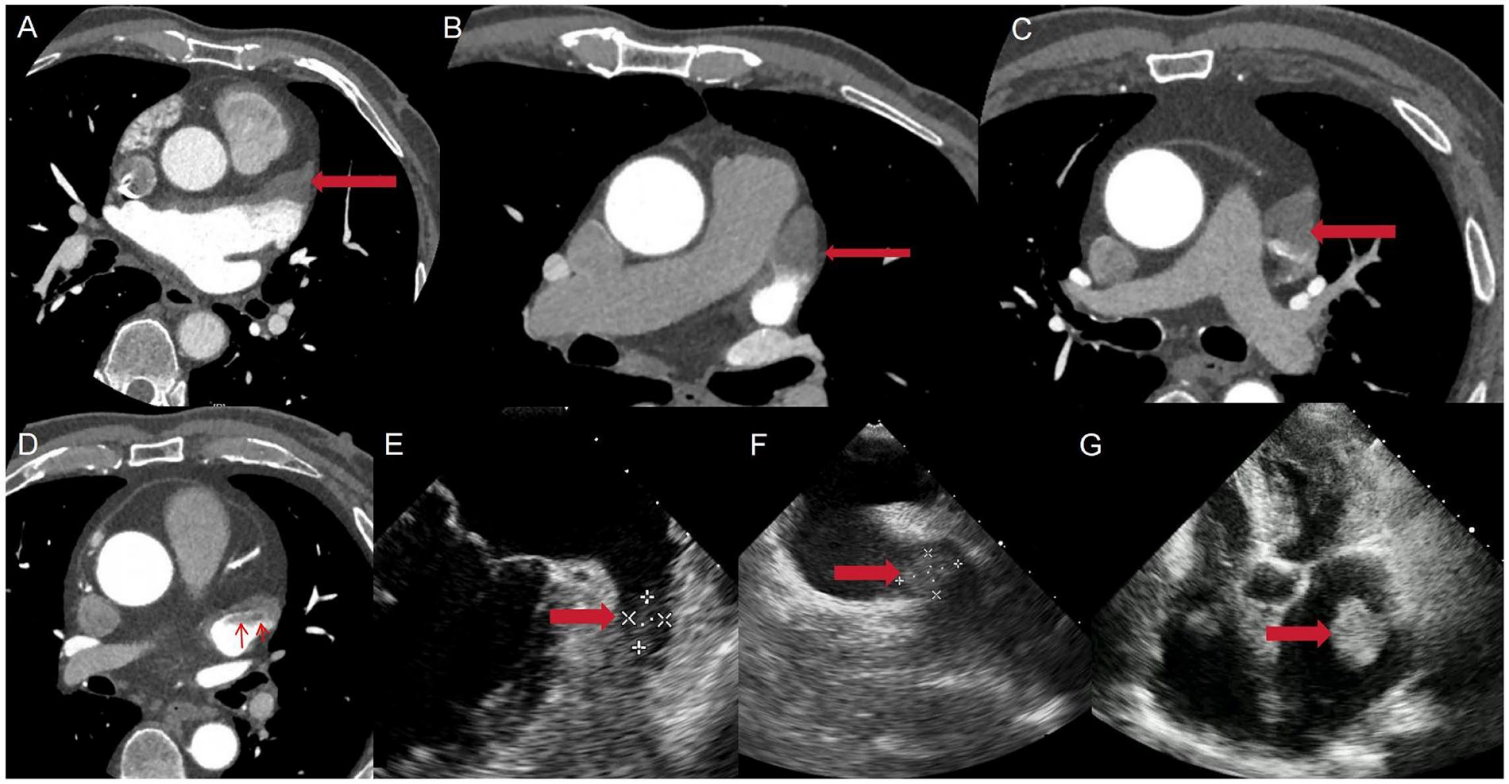
Imaging findings of left atrial appendage (LAA) thrombi. **(A)** CTA of the left atrium showing a thrombus localized to the tip of the LAA and occupying less than half of the interior of the LAA. **(B)** CTA image showing a thrombus occupying more than half of the interior of the LAA but not extending beyond the LAA orifice. **(C)** CTA image demonstrating complete filling of the LAA by the thrombus. **(D)** Image of the same patient as in Panel C, demonstrating a LAA thrombus that extends beyond the LAA orifice and protrudes into the LA. **(E)** TEE reveals thrombus formation within the LAA tip that occupies less than half of the interior of the LAA. **(F)** TEE reveals a thrombus that occupies more than half of the interior of the LAA but not extending beyond the LAA orifice. **(G)** TTE shows a thrombus within the LAA extending into the LA. (The thrombus is indicated by a red arrow in each panel).

Patients were stratified into two groups on the basis of preoperative imaging findings. The Thrombus Group was composed of patients with confirmed or suspected LAA thrombus on preoperative cardiac CTA or TEE/TTE. The No-Thrombus Group was composed of patients with no evidence of LAA thrombus on preoperative imaging. Representative imaging examples of LAA thrombi that defined the thrombus group are provided in Figure 1. These include CTA findings of a thrombus localized to the LAA tip without complete appendage filling (Panel A, B) and a thrombus completely filling the LAA and extending from the LAA orifice into the left atrial cavity (Panel C, D), as well as a TEE/TTE image confirming intracavitary thrombus formation (Panel E, F, G).

### 2.3 Surgical method

The procedure of standalone left atrial appendage clipping is performed with the patient placed in the right lateral decubitus position. Under general anesthesia with double-lumen endotracheal intubation, right-lung ventilation is maintained. The operating port is established at the intersection of the left fourth intercostal space at the mid-axillary line and the left fifth intercostal space at the posterior axillary line. The thoracoscopic port is placed at the intersection of the left sixth intercostal space and the anterior axillary line. Additionally, a 2 cm incision is made at the left seventh intercostal space along the posterior axillary line for the introduction of the atrial appendage clip.

After pericardiotomy, the entire LAA is exposed. Under TEE guidance, the C-Clip® Left Atrial Appendage Closure System (Med-Zenith, Beijing, China) is deployed at the base of the appendage. The clip is released only after the absence of blood flow within the appendage is confirmed by TEE, the residual stump measures less than 1 cm, and no electrocardiographic changes are observed. If preoperative imaging confirms the presence of thrombus within the LAA, the clip must be carefully advanced around the appendage, avoiding compression of the appendage body.

When combined with off-pump coronary artery bypass grafting (OPCAB), the strategy for deploying the LAA clip is similar to that used in the standalone clipping procedure.

### 2.4 Postoperative management

Following LAAC, patients are routinely prescribed oral beta-blockers or amiodarone for ventricular rate control, along with direct oral anticoagulants for three months. For patients with a high bleeding risk (HAS-BLED ≥3) or a history of major bleeding, reduced doses of DOACs are prescribed according to standard dose-reduction criteria. For patients with a history of cerebral infarction (ischemic stroke or TIA), aspirin or clopidogrel is continued as long-term antiplatelet therapy for secondary prevention. Prior to discharge, a follow-up CTA of the left atrium is performed to evaluate the position of the clip and the length of the residual appendage stump, confirm complete closure of the LAA, and assess for any potential dislodgement of the preexisting thrombus.

### 2.5 Clinical follow-up

Patients who underwent LAAC were followed up via outpatient visits and telephone interviews to document the composite thromboembolic events (ischemic stroke, hemorrhagic stroke, or transient ischemic attack) in both the Thrombus and No-Thrombus Groups. All-cause mortality was also recorded for patients in the two groups.

### 2.6 Statistical analysis

Continuous variables are presented as the mean ± standard deviation, and categorical variables are expressed as frequencies and percentages. Between-group comparisons were performed using Student's *t*-test or the Mann‒Whitney *U*-test for continuous variables and the chi-square test or Fisher's exact test for categorical variables, as appropriate. Survival analysis was conducted using the Kaplan‒Meier method. A two-tailed *P* value < 0.05 was considered to indicate statistical significance. All the statistical analyses were conducted using SPSS Statistics version 22.0 (IBM Corp., Armonk, NY, USA) or STATA statistical software version 16.

## 3 Results

### 3.1 Study population and baseline characteristics

A total of 108 patients who underwent epicardial LAAC were included in the final analysis. Patients were stratified into two groups on the basis of preoperative imaging: the Thrombus Group (*n* = 11, 10.2%) and the No-Thrombus Group (*n* = 97, 89.8%).

The baseline demographic and clinical characteristics of the two groups are summarized in Table 1. Patients with LAA thrombus were significantly younger than those without LAA thrombus (60.1 ± 4.1 vs. 66.4 ± 7.3 years; *P* = 0.006). Comorbidity profiles were generally comparable, except for a history of cerebral hemorrhage, which was more frequent in the Thrombus Group (27.3% vs. 6.2%; *P* = 0.016). Accordingly, the Thrombus Group had a significantly higher HAS-BLED bleeding risk score (4.3 ± 1.2 vs. 3.3 ± 1.5; *P* = 0.038), whereas the CHA₂DS₂-VASc stroke risk scores were similar between the groups (4.5 ± 1.0 vs. 4.3 ± 1.4; *P* = 0.768). Echocardiographic parameters, including left atrial size and ejection fraction, were not significantly different. With respect to the surgical approach, a left mini-thoracotomy was utilized significantly more often in the Thrombus Group (18.2% vs. 1.0%; *P* = 0.001). Similarly, concomitant minimally invasive direct coronary artery bypass (MIDCAB) was performed more frequent in this context within the Thrombus Group (18.2% vs. 1.0%; *P* = 0.001). The frequencies of totally thoracoscopic standalone LAAC and concomitant OPCAB were not significantly different between the two cohorts.

**Table 1:** Baseline characteristics of the study population.

Variables	Thrombus group (*n* = 11)	No-thrombus group (*n* = 97)	*P* value
Age, years	60.1 ± 4.1	66.4 ± 7.3	0.006
Male, *n* (%)	10 (90.9)	70 (72.2)	0.179
Type of AF, *n* (%)			
Paroxysmal	1 (9.1)	23 (23.7)	0.269
Persistent	9 (81.8)	55 (56.7)	0.199
Permanent	1 (9.1)	19 (19.6)	0.396
Comorbidities, *n* (%)			
Hypertension	9 (81.8)	62 (63.9)	0.325
Diabetes Mellitus	3 (27.3)	28 (28.9)	0.912
Stroke/TIA	9 (81.8)	56 (57.7)	0.194
Cerebral Hemorrhage	3 (27.3)	6 (6.2)	0.016
Coronary Heart Disease	5 (45.5)	61 (62.9)	0.261
Scores			
CHA₂DS₂-VASc	4.5 ± 1.0	4.3 ± 1.4	0.768
HAS-BLED	4.3 ± 1.2	3.3 ± 1.5	0.038
Echocardiography			
Left Atrial Size, mm	42.6 ± 6.8	40.8 ± 6.7	0.408
Ejection Fraction, %	59.5 ± 3.8	61.4 ± 5.8	0.315
Approach, *n* (%)			
Totally thoracoscopic	9 (81.8)	91 (93.8)	0.405
Median sternotomy	0 (0)	5 (5.2)	0.989
Left mini-thoracotomy	2 (18.2)	1 (1.0)	0.001
Concomitant procedure, *n* (%)			
Standalone	9 (81.8)	91 (93.8)	0.405
OPCAB	0 (0)	5 (5.2)	0.989
MIDCAB	2 (18.2)	1 (1.0)	0.001

Data are presented as the mean ± SD, *n* (%). TIA, transient ischemic attack. OPCAB, off-pump coronary artery bypass grafting. MIDCAB, minimally invasive direct coronary artery bypass.

### 3.2 Intraoperative complications of LAAC

Intraoperative safety outcomes are detailed in Table 2. No major intraoperative complications were observed in either patient cohort. Specifically, there were zero occurrences of stroke, death, conversion to mini-sternotomy or mini-thoracotomy for bleeding, procedure discontinuation due to bleeding, or phrenic nerve injury among all 108 patients. Consequently, the total number of intraoperative complications was 0 (0.0%) in both the Thrombus Group and the No-Thrombus Group.

**Table 2:** Intraoperative complications of LAAC.

Variables, *n* (%)	Thrombus group (*n* = 11)	No-thrombus group (*n* = 97)
Stroke	0 (0.0)	0 (0.0)
Death	0 (0.0)	0 (0.0)
Mini-sternotomy for bleeding	0 (0.0)	0 (0.0)
Mini-thoracotomy for bleeding	0 (0.0)	0 (0.0)
Bleeding with discontinuation of procedure	0 (0.0)	0 (0.0)
Phrenic nerve injury	0 (0.0)	0 (0.0)
Total number of intraoperative complications	0 (0.0)	0 (0.0)

Data are presented as *n* (%).

### 3.3 Postoperative complications of LAAC

Postoperative complication rates within 30 days were low in both patient groups, with no statistically significant differences observed between patients with and without a preoperative LAA thrombus, as detailed in Table 3.

**Table 3:** Postoperative complications of LAAC.

Variables, *n* (%)	Thrombus group (*n* = 11)	No-thrombus group (*n* = 97)	*P* value
Major			
Clip-related complications	0 (0.0)	0 (0.0)	
Death	0 (0.0)	0 (0.0)	
Pericardial effusion or tamponade	0 (0.0)	0 (0.0)	
Hemothorax	0 (0.0)	0 (0.0)	
Empyema	0 (0.0)	0 (0.0)	
Reintubation	0 (0.0)	0 (0.0)	
Pulmonary embolism	0 (0.0)	0 (0.0)	
Permanent phrenic nerve injury	0 (0.0)	0 (0.0)	
Stroke	0 (0.0)	1 (1.0)	0.735
Transient ischemic attack	0 (0.0)	1 (1.0)	0.735
Cerebral Hemorrhage	0 (0.0)	0 (0.0)	
Atrioesophageal fistula	0 (0.0)	0 (0.0)	
Myocardial infarction	0 (0.0)	0 (0.0)	
Minor			
Permanent pacemaker implantation	0 (0.0)	0 (0.0)	
Pericardial fluid	0 (0.0)	0 (0.0)	
Pneumothorax	0 (0.0)	1 (1.0)	0.735
Pleural effusion	1 (9.1)	4 (4.1)	0.457
Hemothorax	0 (0.0)	0 (0.0)	
Urinary tract infection	0 (0.0)	0 (0.0)	
Wound infection	0 (0.0)	0 (0.0)	
Pulmonary Infection	0 (0.0)	1 (1.0)	0.735
Gastrointestinal bleeding	0 (0.0)	0 (0.0)	

Data are presented as *n* (%).

In the Thrombus Group (*n* = 11), only one minor complication was recorded: a single case of pleural effusion (9.1%). No major complications occurred. In the No-Thrombus Group (*n* = 97), complications were also infrequent. One patient (1.0%) experienced a stroke, one (1.0%) had a transient ischemic attack, one (1.0%) developed a pneumothorax, one (1.0%) was diagnosed with a pulmonary infection, and four patients (4.1%) had pleural effusion. Overall, the presence of a preoperative LAA thrombus was not associated with a significant increase in the incidence or severity of early postoperative complications following epicardial LAAC.

### 3.4 Postoperative CTA of left atrium

Postoperative CTA was performed in all patients before discharge to evaluate the procedural results. As shown in Table 4, no residual flow was observed in the Thrombus Group, whereas one patient (1.0%) in the No-Thrombus Group had residual flow. A residual LAA stump >1 cm was noted in one patient (9.1%) in the Thrombus Group and in three patients (3.1%) in the No-Thrombus Group (*P* = 0.876). No thrombus dislodgement was detected on postoperative CTA in either group.

**Table 4:** Postoperative CTA of LA.

Variables, *n* (%)	Thrombus group (*n* = 11)	No-thrombus group (*n* = 97)	*P* value
LAA residual flow	0 (0.0)	1 (1.0)	0.999
LAA stump > 1 cm	1 (9.1)	3 (3.1)	0.876
Thrombus dislodgement	0 (0.0)	0 (0.0)	

Data are presented as *n* (%).

### 3.5 Cerebrovascular events and all-cause mortality

**Figure 2:**
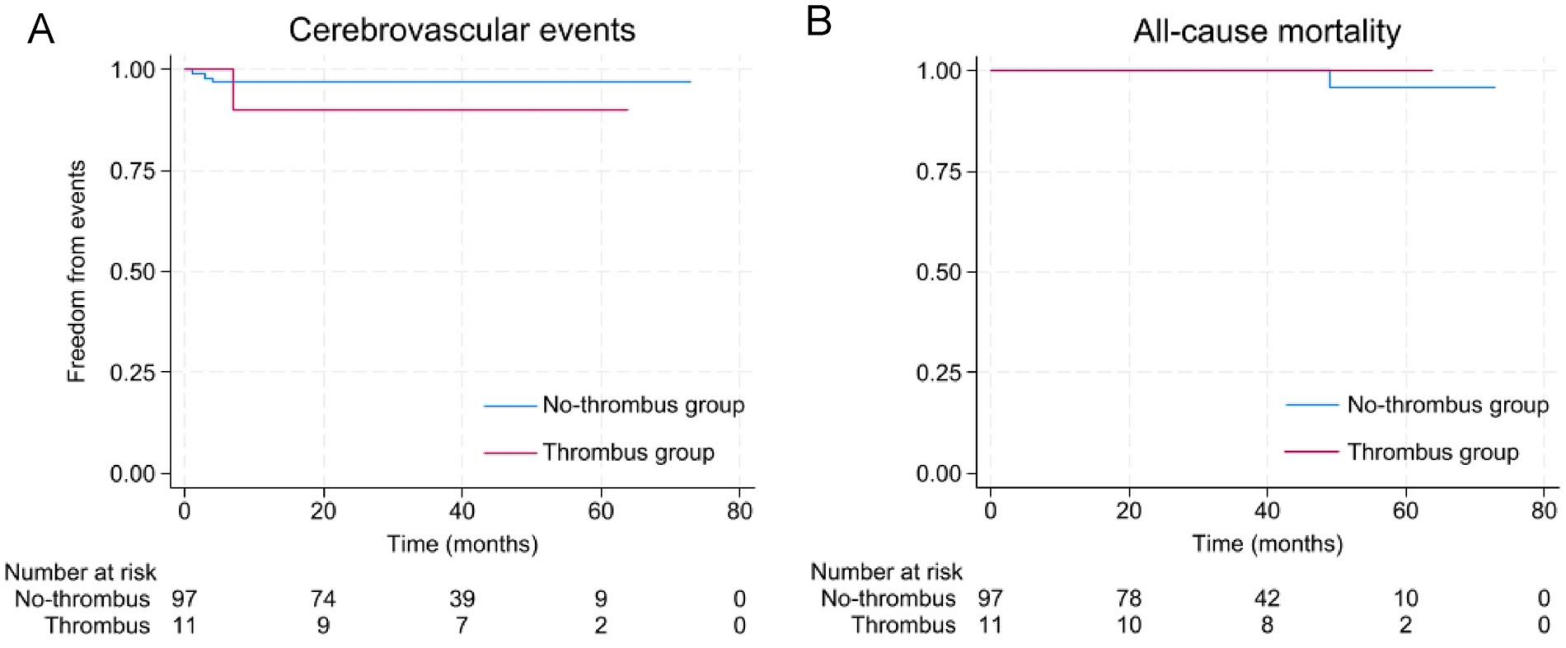
Kaplan–Meier analysis of LAAC outcomes. **(A)** Freedom from ischemic stroke, hemorrhagic stroke, or transient ischemic attack, *P* = 0.334. **(B)** Freedom from all-cause mortality, *P* = 0.597.

The long-term efficacy and safety of epicardial LAAC in both patient groups were evaluated via Kaplan‒Meier survival analysis, as illustrated in Figure 2. The median follow-up duration in the No-Thrombus Group was 38 months (interquartile range, 24–49 months), and in the Thrombus Group, the median follow-up duration was 53 months (interquartile range, 36–54 months). During a total follow-up of 294.3 patient-years in the No-Thrombus Group, the observed cerebrovascular event rate was low at 1.0 per 100 patient-years, and the all-cause mortality was 0.3 per 100 patient-years; moreover, during a total follow-up of 42.1 patient-years in the Thrombus Group, the observed cerebrovascular event rate was 2.3 per 100 patient-years, and no death was observed. Notably, the event-free survival from a composite of thromboembolic events (ischemic stroke, hemorrhagic stroke, or transient ischemic attack) was not significantly different between patients with and without a preoperative LAA thrombus (log-rank *P* = 0.334). Furthermore, the analysis of all-cause mortality also revealed comparable survival curves between the two groups, with no statistical significance reached (log-rank *P* = 0.597).

## 4 Discussion

In patients with non-valvular AF, more than 90% of left atrial thrombi originate from the LAA (16). During AF, the pouch-like structure of the LAA promotes blood stasis, thereby increasing thrombus risk compared with individuals in sinus rhythm (17). Unlike the smooth endocardial surface of the left atrium, the interior of the LAA is predominantly lined with pectinate muscles (18). The cauliflower-like structure of the LAA, characterized by a larger orifice and slower blood flow, is associated with a higher stroke risk (19). Thrombi most commonly form at the LAA apex, likely because of pronounced blood stagnation in this blind-ended region (20). Once formed, thrombi tend to adhere relatively firmly to the pectinate muscles, making them less prone to embolization. However, when the thrombus extends toward the LAA orifice, the smoother endocardial surface near the left atrium provides less anchorage, thereby increasing the risk of detachment and subsequent systemic embolism.

Current guidelines recommend anticoagulation therapy, such as warfarin or direct oral anticoagulants, for AF patients with a CHA_2_DS_2_-VASc score ≥ 2 (21). However, for patients with a HAS-BLED score > 3, anticoagulant treatment is associated with an increased risk of bleeding (22). Some individuals, particularly those with a history of major bleeding, are intolerant to long-term anticoagulant treatment. Therefore, for this specific group of AF patients with LAA thrombus and a high bleeding risk, alternative therapies are urgently needed.

In this context, epicardial LAAC has emerged as a valuable nonpharmacological alternative to anticoagulant therapy and is gaining increasing clinical attention and application. The LAA is surgically closed in LAAC, thereby anatomically eliminating the nidus for thrombus formation and effectively reducing the risk of thromboembolic events (23, 24). Compared with traditional anticoagulant therapy, its greatest advantage lies in eliminating the need for long-term anticoagulant medication, fundamentally avoiding drug-related bleeding risks. This is particularly suitable for AF patients with high HAS-BLED scores, contraindications to anticoagulant therapy, or intolerance to anticoagulant therapy. The procedure can typically be performed either via a totally thoracoscopic approach or concurrently during cardiac surgery, resulting in relatively minimal trauma, straightforward operation, and rapid postoperative recovery (25). For patients with existing thrombi located at the appendage apex, epicardial LAAC directly obstructs blood flow within the appendage, preventing further thrombus growth and dislodgement, thus providing more direct and durable protection for patients. Multiple clinical studies have demonstrated that compared with long-term anticoagulant therapy, successful LAAC can significantly reduce the incidence of stroke in AF patients, with a notably lower rate of postoperative bleeding complications (9). Therefore, for this specific group of AF patients with LAA thrombi and a high bleeding risk, epicardial LAAC undoubtedly offers a crucial therapeutic pathway. It has the potential to effectively prevent stroke while maximizing patient safety and improving long-term prognosis, thereby addressing significant unmet clinical needs.

In this retrospective study, the safety and outcomes of epicardial LAAC in patients with a preoperatively identified LAA thrombus were evaluated. The principal finding is that the presence of an LAA thrombus was not associated with a significant increase in perioperative complications or adverse long-term clinical outcomes compared with patients without a thrombus. These findings suggest that, with careful patient selection, epicardial LAAC may represent a feasible and safe alternative for individuals with LAA thrombi who are unsuitable for long-term anticoagulant therapy.

Notably, the traditional view of the presence of a LAA thrombus as a contraindication to LAAC requires nuanced reconsideration in the surgical context. Unlike percutaneous approaches, where device delivery necessitates traversing the left atrium and engaging the LAA orifice—maneuvers that inherently risk thrombus dislodgement—surgical exposure offers distinct advantages (26). When the left atrium is opened during concomitant cardiac procedures, the LAA can be directly inspected, and any thrombus can be aspirated or surgically removed under direct vision before closure (27). However, in totally thoracoscopic standalone left atrial appendage closure, which is performed without cardiopulmonary bypass or the ability to open the LAA for thrombus evacuation, the presence of a thrombus has traditionally been considered a contraindication for epicardial LAAC (28).

Contrary to the prevailing concern regarding thrombus dislodgement and periprocedural embolism, no intraoperative strokes or systemic embolic events occurred in the Thrombus Group in our study. Early postoperative morbidity was low and comparable between groups, with the observed complications consistent with the general risk profile of thoracic cardiac surgery rather than being uniquely attributable to thrombus presence. These findings indicate that, with meticulous surgical techniques, the procedural risk in selected patients with thrombi does not appear to be elevated.

### 4.1 The importance of thrombus morphology and risk stratification

**Figure 3:**
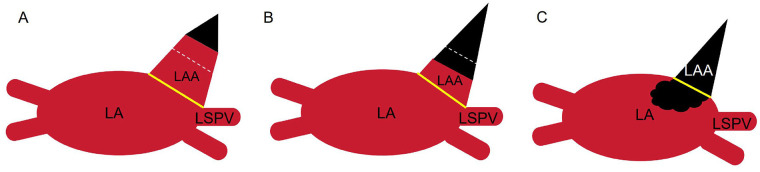
Classification of LAA thrombi. **(A)** Type I: Thrombus confined to the tip of the LAA, occupying less than half of its volume (white dashed line). **(B)** Type II: Thrombus extends beyond half of the LAA volume but does not reach the LAA orifice (yellow line). **(C)** Type III: Thrombus extends beyond the LAA orifice into the left atrial cavity. (The thrombus is indicated by a black triangle in each panel. LA, Left atrium; LSPV, left superior pulmonary vein. The solid yellow line indicates the LAA orifice, and the white dashed line indicates the virtual midpoint of the LAA volume.).

Our analysis revealed that thrombus burden and location are critical determinants of procedural risk. The proposed imaging-based classification (Figure 3) provides a practical framework for clinical decision-making:

Type I thrombi are confined to the tip of the LAA, occupying less than half of the LAA volume. This morphology (*n* = 11) is likely associated with the lowest embolic risk during manipulation. Our favorable outcomes demonstrate that epicardial LAAC is safe and feasible in this specific population.

Type II thrombi extend from the tip into the body of the LAA, exceeding half of the LAA volume but not reaching the orifice. This type of thrombus is associated with intermediate risk. Surgery requires cautious consideration and should only proceed after thorough multidisciplinary evaluation, balancing individual thromboembolic and bleeding risks.

Type III thrombi fill the entire LAA and extend beyond the orifice into the left atrial cavity. This thrombus type is associated with the highest risk due to its mobility and location. Epicardial LAAC is not recommended for these patients, and alternative management strategies are imperative.

This morphology-driven approach advocates for a nuanced, individualized strategy over a binary contraindication, enabling refined risk stratification.

### 4.2 Risk stratification: thrombus morphology and device interaction

The proposed stratification is underpinned by distinct anatomical and mechanical considerations. For Type I thrombi confined to the LAA tip, the risk of dislodgement is relatively low because the epicardial clip is applied at the thrombus-free base, minimizing direct manipulation. Moreover, apical thrombi are often firmly embedded within the trabeculated pectinate muscles. Crucially, successful clip deployment entraps and physically isolates the thrombus within the excluded appendage, eliminating its communication with the systemic circulation. In contrast, Type II thrombi, which occupy a large volume of the LAA, pose a greater theoretical risk. Clip closure may mechanically compress the LAA body, potentially displacing or fragmenting the thrombus proximally toward the left atrium; extreme caution should be taken to avoid this “squeezing effect”. Finally, for Type III thrombi, any mechanical manipulation has a high risk of fragmenting the mobile intra-atrial clot, and the presence of this type of thrombus is a contraindication for epicardial LAAC.

Given the potential risks associated with manipulating a thrombus-containing LAA, adherence to meticulous surgical techniques is paramount when performing clip closure in this context. The cornerstone of safe epicardial closure in patients with a preexisting thrombus is gentle manipulation of the LAA to avoid any unintended compression of the thrombus-containing body, which could theoretically provoke fragmentation or dislodgement. To this end, intraoperative TEE guidance is indispensable, serving not only to confirm the diagnosis but also, more critically, to precisely identify a “thrombus-free zone” at the base of the LAA before clip deployment. This imaging confirmation allows the surgeon to plan the optimal landing zone. The fundamental surgical technique involves deploying the clip at the extreme base of the LAA, thereby achieving complete exclusion of the appendage and its thrombotic contents from the systemic circulation while ensuring that the clip itself avoids any direct contact with the thrombus. On the basis of our experience, particularly with Type I thrombi, we advocate for a stepwise approach to maximize safety: (1) complete and atraumatic thoracoscopic exposure of the entire LAA; (2) identification of the thrombus-free landing zone at the base using intraoperative TEE; (3) careful advancement and positioning of the C-Clip® around the identified base, ensuring no squeezing or manipulation of the distal appendage body; and (4) final confirmation with TEE of complete closure with no residual flow within the appendage stump. Adherence to this protocol was, in our view, instrumental in achieving zero intraoperative embolic events in our thrombus cohort.

The C-Clip® Left Atrial Appendage Closure System, deployed via a transepicardial approach under thoracoscopic guidance, offers distinct advantages over percutaneous endocardial devices for patients with a preexisting LAA thrombus, as summarized in Table 5.

**Table 5:** Comparison of epicardial vs. percutaneous LAAC indications in patients with a LAA thrombus.

Feature	Thoracoscopic surgical LAAC (Epicardial clipping)	Percutaneous LAAC (Endocardial device)
Suitable Candidates	AF patients with high bleeding risk who are intolerant to anticoagulant treatment	AF patients without a LAA thrombus who are unsuitable for long-term anticoagulant treatment
Approach	Epicardial	Endocardial
LAA Thrombus Status	Feasible for Type I (tip-confined) thrombi	Absolute contraindication regardless of thrombus type
Device Interaction with Thrombus	None; the clip interacts with the LAA externally without contacting the thrombus	Device must interact with the LAA internally, risking thrombus disruption

Unlike percutaneous LAAC, where delivery sheaths and closure devices must traverse the left atrium and engage the LAA orifice—which has inherent risks of thrombus dislodgement during intracavitary manipulation—the C-Clip® system applies an epicardial clip at the external base of the appendage, completely avoiding manipulation of the thrombus-containing cavity. Thoracoscopic surgery provides direct visual confirmation of LAA anatomy, allowing surgeons to identify the thrombus-free zone and avoid compression of the thrombus-filled apex, whereas percutaneous approaches rely on indirect fluoroscopic guidance that cannot reliably distinguish thrombi from trabeculations (29). Furthermore, the epicardial LAAC achieves complete extrinsic exclusion of the LAA, physically isolating the thrombus from the systemic circulation without any intra-appendage hardware (30), in contrast to percutaneous devices that are implanted within the LAA cavity and may disturb the thrombus during positioning or leave components in contact with residual thrombus. These technical advantages—external exclusion without intracavitary manipulation—explain why percutaneous LAAC guidelines uniformly list the presence of a LAA thrombus as a contraindication, while our findings suggest that for carefully selected patients with Type I thrombus, the epicardial approach offers a viable therapeutic alternative. By deploying the clip at the thrombus-free base and capitalizing on the anatomical fact that apical thrombi are firmly embedded in pectinate muscles and remote from the closure site, the epicardial LAAC achieves the favorable safety profile observed in our study, with no intraoperative strokes or embolic events observed in the Thrombus group.

Our results contribute to a growing, yet limited, body of evidence re-evaluating the prohibition of intervention in AF patients with a LAA thrombus. While prior case reports have described successful procedures (31), robust comparative data are scarce. This study provides a direct comparative analysis, strengthening the argument against an absolute contraindication in the surgical context. This contrasts sharply with percutaneous LAA closure, where the presence of a thrombus remains a firm exclusion criterion owing to the inherent limitations of device-based, nonvisualized techniques. The surgical approach offers a distinct potential advantage: direct visualization, the possibility for controlled thrombus management, and epicardial clip placement, which may collectively mitigate embolic risk. However, recently, Katz et al. reported that in patients undergoing percutaneous LAAC, the presence of a distal LAA thrombus was not associated with worse long-term outcomes (32). Our epicardial LAAC findings are in line with these results, showing no significant differences in thromboembolic events or mortality between thrombus and no-thrombus groups. These data suggest that both percutaneous and epicardial LAAC may be feasible in selected patients with LAA thrombus, although the procedural techniques and exclusion mechanisms differ.

### 4.3 Study limitations

Several limitations of this study should be acknowledged. The retrospective, single-center design may introduce selection bias and limit the generalizability of our findings to other institutions or populations with different patient demographics, surgical expertise, or perioperative management protocols. The relatively small number of patients in the Thrombus Group (*n* = 11), although reflecting the rarity of such cases in clinical practice, reduces statistical power and precludes robust subgroup analyses or definitive conclusions regarding the safety and efficacy of LAAC in patients with more extensive thrombus burdens (e.g., Type II or III). Consequently, our findings are primarily applicable to patients with Type I thrombi, and caution is warranted when these results are extrapolated to those with higher-risk thrombus morphologies.

The follow-up duration, while adequate for assessing perioperative safety and midterm outcomes, may be insufficient to capture very late thromboembolic events or device-related complications. Longer-term surveillance is necessary to confirm the durability of stroke prevention and the absence of late thrombus recurrence or dislodgement.

In addition, the decision to proceed with surgery was made by a multidisciplinary heart team, introducing a potential selection bias toward patients with more favorable thrombus characteristics (i.e., Type I) and lower overall surgical risk. This may have positively influenced the observed outcomes and limits the applicability of our results to unselected patient populations.

Although a morphology-based classification system (Types I–III) was proposed to guide patient selection, this system has not yet been externally validated. Its reproducibility across different imaging modalities and interpreters remains to be established. In future studies, the standardization of this classification system should be a focus and its interobserver variability should be assessed.

These limitations highlight the need for prospective, multicenter registries with larger, more diverse cohorts and standardized imaging protocols to validate our findings, refine risk stratification, and define the optimal role of epicardial LAAC in patients with a LAA thrombus and high bleeding risk.

## 5 Conclusion

In conclusion, this study suggests that epicardial LAAC can be performed safely in carefully selected patients with a preoperative LAA thrombus without compromising perioperative safety or long-term efficacy. Thrombus morphology appears to be a predictor of procedural safety. These findings suggest a paradigm shift from a blanket contraindication to a morphology-guided, heart-team-based decision-making process.

Future research should include prospective, multicenter registries with larger cohorts to validate this risk stratification model. Efforts should focus on standardizing the imaging classification of LAA thrombi, defining optimal surgical techniques for each subtype, and establishing evidence-based protocols for managing high-bleeding-risk patients with coexisting LAA thrombi.

## Data Availability

The original contributions presented in the study are included in the article/Supplementary Material, further inquiries can be directed to the corresponding author.
